# Poor predictability of QuantiFERON-TB assay in recipients and donors for tuberculosis development after kidney transplantation in an intermediate-TB-burden country

**DOI:** 10.1186/s12882-017-0506-9

**Published:** 2017-03-14

**Authors:** Enkthuya Jambaldorj, Miyeun Han, Jong Cheol Jeong, Tai Yeon Koo, Sang Il Min, Eun Young Song, Jongwon Ha, Curie Ahn, Jaeseok Yang

**Affiliations:** 1 Transplantation Research Institute, Seoul National University Hospital, 101 Daehak-ro, Jongno-gu, Seoul, 110-744 Republic of Korea; 2 Department of Internal Medicine, Seoul National University College of Medicine, Seoul, Korea; 3 Department of Internal Medicine, Ajou University School of Medicine, Suwon, Korea; 4 Transplantation Center, Seoul National University Hospital, Seoul, Korea; 5 Department of Surgery, Seoul National University College of Medicine, Seoul, Korea; 6 Department of Laboratory Medicine, Seoul National University College of Medicine, Seoul, Korea

**Keywords:** Donor, Kidney transplantation, Recipient, Tuberculosis

## Abstract

**Background:**

Tuberculosis (TB) is a common opportunistic infection after kidney transplantation (KT). The QuantiFERON-TB-Gold In-Tube test (QFT) is widely used for assessing latent TB; however, it is currently unclear whether the pre-KT QFT of the recipient and donor can predict post-KT TB.

**Methods:**

We retrospectively reviewed patients who received KT between January 2009 and December 2015 at Seoul National University Hospital. The QFT was performed in 458 KT recipients and 239 paired living donors, and 138 KT recipients underwent both the QFT and tuberculin skin test (TST). After excluding 12 patients diagnosed as having clinically latent TB, we evaluated whether the QFT of the recipient and donor was predictive for new-onset active TB after KT.

**Results:**

The QFT was positive in 101 (22.1%) recipients and associated with clinically latent TB before KT (*P* < 0.05). However, agreement between the TST and QFT was poor (κ = 0.327). Post-KT TB occurred in 1 of 95 recipients with a positive QFT, and 2 cases of TB occurred among 351 patients with a negative or indeterminate QFT. The incidence of TB was 242 cases/100,000 person-years among 446 KT recipients with a median follow-up of 30.2 months. The QFT of recipients could not predict post-KT TB in Poisson regression analysis (relative risk [RR], 1.847; 95% confidence interval [CI], 0.168–20.373; *P* = 0.616). Of 234 living donor-recipient pairs, the QFT of the recipient (RR, 5.012; 95% CI, 0.301–83.430; *P* = 0.261) and QFT of the donor (RR, 1.758; 95% CI, 0.106–29.274; *P* = 0.694) could not predict post-KT TB.

**Conclusion:**

The QFT of recipients or living donors pre-KT cannot predict the short-term development of post-KT TB in an intermediate TB-burden country.

## Background

Tuberculosis (TB) is a common opportunistic infection after transplantation. The incidence of TB among transplant recipients is 20–74 times higher than that in the general population [1], and TB after transplantation can adversely affect graft viability and patient survival [2]. The most common cause of TB in kidney transplantation (KT) recipients is reactivation of latent TB. Therefore, screening and treating active or latent TB in all transplant candidates is widely recommended [2–4].

History taking, physical examination, and chest radiography should be performed to assess for TB before transplantation. To make a diagnosis of latent TB, the tuberculin skin test (TST) or interferon (IFN)-γ release assay (IGRA) is used. However, the Korean guidelines for TB, which were revised in 2014, recommend the use of IGRA alone or TST combined with IGRA for diagnosing latent TB in patients with end-stage renal disease (ESRD), because TST may yield a false positive result in those with a previous Bacillus Calmette-Guérin (BCG) vaccination or a false negative in patients with ESRD [5, 6].

The TB burden of South Korea is decreasing, but the country is still classified as having an intermediate TB burden, with an annual TB incidence of 86/100,000 person-years in 2015 [7] and a TB incidence after KT of 0.283 cases/100 patient-years in a previous study [8]. The QuantiFERON-TB-Gold In-Tube test (QFT) (Cellestis Ltd., Carnegie, Victoria, Australia) was introduced at our center in 2009. However, because the significance of QFT results in immunosuppressed patients was not sufficiently validated, we did not administer anti-TB medications to patients based on the results of QFT alone, unless there was evidence of clinically latent TB, which includes abnormal chest radiograph findings or a history of TB without complete anti-TB medication and recent close contact with individuals with active TB. The predictive ability of the QFT for post-KT TB in KT recipients is still unclear; therefore, we investigated the efficacy of using the QFT pre-KT in recipients and donors to predict the subsequent development of TB after KT.

## Methods

### Study population

This study was a retrospective, single-center study. Nine hundred seventy-seven cases of KT were performed from January 1, 2009 to December 31, 2015 at Seoul National University Hospital. KT recipients who underwent the QFT were enrolled in this study. The association between clinical latent TB and QFT or TST was analyzed with the recipient who underwent the QFT and TST. The predictability of the QFT of recipients and donors for post-KT TB was analyzed after excluding recipients with isoniazid prophylaxis. This study was approved by our institutional review board (H-1310-069-527), and the need for informed consent was waived because of its retrospective design.

### Data collection

The demographic characteristics and laboratory results of KT recipients and donors were retrospectively obtained from medical records. Age, gender, body weight, KT time, donor type, donor age, donor gender, comorbid disease including a history of TB infection and previous BCG vaccination, post-transplant medications including immunosuppressants, and laboratory data including QFT and chest radiographs were collected. TB development after KT was determined by microbiological confirmation and the initiation of anti-TB medication. Clinical latent TB was defined as (1) an inadequate history of anti-TB medication with a history of TB infection or an inactive TB lesion on a chest radiograph, (2) close contact with a person with pulmonary TB within the past year, and (3) recent conversion of the TST to a positive status [9, 10].

### Laboratory testing (IGRAs and TST)

Peripheral blood samples for the QFT were collected, transferred to the laboratory, and processed within 3 h according to the manufacturer’s instructions [11]. The QFT results were classified as positive, negative, or indeterminate, as previously described : positive if the response to the specific antigens was ≥ 0.35 IU/mL, regardless of the value of the positive control; negative if the response to the specific antigens was < 0.35 IU/mL and the IFN-γ level of the positive control was ≥ 0.5 IU/mL; and indeterminate if both antigen-stimulated samples were < 0.35 IU/mL and the value of the positive control was < 0.5 IU/mL [12].

TST was performed by trained nurses within 1 week after the QFT. Two tuberculin units of purified protein derivative RT23 (Statens Serum Institute, Copenhagen, Denmark) were injected intradermally on the volar side of the forearm contralateral to the patient’s vascular access (the Mantoux technique). The diameter of induration was measured 48 h after administration. The test was considered positive if the diameter of induration was ≥10 mm.

### Statistical analysis

Categorical variables are presented as frequencies, and continuous values are expressed as the mean ± standard deviation. Proportions were compared using the chi-square test, and continuous variables were compared using the Student t-test. Poisson regression models were used to estimate the relative risk (RR) and 95% confidence interval (CI). A P-value <0.05 was considered statistically significant. All statistical analyses were conducted using SPSS, version 22.0 (SPSS Inc., Chicago, IL).

## Results

### Characteristics of the study population

The QFT was performed in 458 KT recipients (living donor KT, 303; deceased donor KT, 155). Baseline characteristics for the 458 recipients are shown in Table 1. Patients’ mean age was 44.5 ± 15.4 years, and 300 (65.4%) were men. Twenty-one (4.6%) KT recipients had a TB history, and all of them received adequate anti-TB medication. Seventeen (3.7%) patients showed radiologic evidence of previous TB, such as a calcified granuloma or nodule (six patients), nodular opacity (8), and other (3). However, 12 had not received adequate anti-TB medication. Hypertension and diabetes were found in 377 (82.3%) and 110 (24.0%) patients, respectively.

**Table 1:** Baseline clinical characteristics of study population

	Recipients (*n* = 458)	Recipient-donor pairs (*n* = 239)	
		Recipients (*n* = 239)	Donors (*n* = 239)
Age, year (± SD)	44.5 ± 15.4	40.6 ± 16.1	45.9 ± 10.2
Male gender	300 (65.5)	157 (65.7)	103 (43.1)
History of TB infection	21 (4.6)	7 (2.9)	19 (7.9)
History of close contact with active TB	0 (0.0)	0 (0.0)	0 (0.0)
Abnormal chest radiography	17 (3.7)	7 (2.9)	14 (5.9)
History of adequately treated TB	5 (1.1)	2 (0.8)	1 (0.4)
History of untreated TB	12 (2.6)	5 (2.1)	13(5.4)
Underlying disease			
Hypertension	377 (82.3)	184 (77.0)	19 (7.9)
Diabetes mellitus	110 (24.0)	49 (20.5)	1 (0.4)
History of malignancy	32 (7.0)	9 (3.8)	0 (0.0)
COPD	3(0.7)	1(0.4)	0 (0.0)
Laboratory findings			
WBC count (cells/μL)	6850.6 ± 3617.4	7004.2 ± 3633.4	6175.2 ± 1765.9
Albumin (g/dL)	4.0 ± 1.6	3.9 ± 0.6	4.4 ± 0.4
Cholesterol (mg/dL)	160.4 ± 75.9	159.6 ± 97.9	197.9 ± 61.6

*COPD* chronic obstructive pulmonary disease, *SD* standard deviation, *TB* tuberculosis, *WBC* white blood cells

Of 458 recipients with QFT results, 239 matched living donors underwent the QFT. Clinical information for the 239 paired recipients and living donors are shown in Table 1. The mean age of the 239 recipients was 40.6 ± 16.1 years, and 157 (65.7%) were men. Seven (2.9%) had a history of TB, and seven (2.9%) had inactive TB lesions on a chest radiograph. The mean age of the 239 paired donors was 45.9 ± 10.2 years, and 103 (43.1%) were men. Nineteen (7.9%) donors had a history of TB, and 14 (5.9%) had inactive TB lesions on a chest radiograph. Nineteen (7.9%) donors had hypertension and 1 (0.4%) had diabetes mellitus.

### Association of the diagnostic tests with clinically latent TB infection

Of 458 recipients with QFT results, 138 also underwent TST. The association of the diagnostic tests with clinically latent TB was analyzed in 138 recipients who underwent both tests (Table 2). Among 458 KT recipients with QFT results, 101 (22.1%) showed positive QFT results, and a positive QFT result was associated with radiologic evidence of previous TB (*P* < 0.001), a history of previous TB (*P* < 0.05), and overall clinically latent TB (*P* < 0.05).

**Table 2:** The association between Tuberculin skin test or QuantiFERON results and latent tuberculosis

	QuantiFERON (*n* = 458)		QuantiFERON (*n* = 138)		TST ≥ 10 mm (*n* = 138)	
	Positive	Negative/indeterminate	Positive	Negative/indeterminate	Positive	Negative
Number (percent, %)	101 (22.1)	357 (77.9)	36 (26.1)	102 (73.9)	18 (13.0)	120 (87.0)
Abnormal chest radiography	10 (9.9)	7 (2.0)**	4 (11.1)	3 (2.9)	0 (0.0)	7 (5.8)
History of previous TB	10 (9.9)	11 (3.1)*	3 (8.3)	3 (2.9)	2 (11.1)	4 (2.9)
Clinical latent TB	6 (5.9)	6 (1.7)*	3 (8.3)	3 (2.9)	0 (0.0)	6 (5.0)

*TB* tuberculosis, *TST* tuberculin skin test

*P < 0.05, **P < 0.001 by Chi-square test

Among 138 KT recipients who underwent QFT and TST, the QFT tended to be associated with a chest radiographic lesion, history of previous TB, and clinically latent TB despite the absence of statistical significance (Table 2). However, TST was not associated with clinically latent TB, and agreement between TST and QFT in these 138 KT recipients was poor (κ = 0.327).

### Observations on the development of TB after KT

**Fig. 1:**
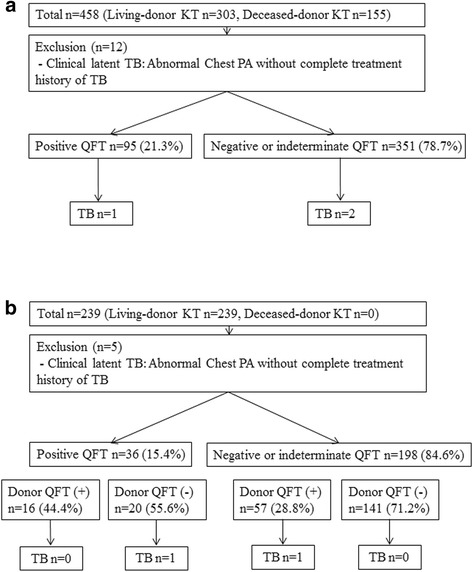
The development of TB after KT. **a** Four hundred fifty-eight KT recipients who underwent a QFT. **b** Two hundred thirty-nine KT recipients with a paired living donor with a QFT. KT, kidney transplantation; TB, tuberculosis; QFT, QuantiFERON-TB-Gold In-Tube test

After excluding 12 patients with clinically latent TB who received isoniazid treatment, 446 cases were analyzed to determine whether QFT was predictive for post-KT TB development. The predictive ability of QFT for post-KT TB development was also analyzed in 234 living donor-recipient pairs after excluding 5 recipients with clinically latent TB who received isoniazid treatment (Fig. 1a and b).

Active TB occurred in 3 of 446 KT recipients, 1 with a positive QFT result and 2 with negative or indeterminate results, at a median follow-up of 30.2 months (Fig. 1a). The incidence of TB was estimated to be 0.242/100 person-years; 0.360/100 person-years in the positive QFT group and 0.208/100 person-years in the negative or indeterminate QFT group (Table 3).

**Table 3:** Tuberculosis incidence according to donor/recipient QuantiFERON status

Variables	TB incidence rates				
	No. of patients	No. of TB cases	No. of person-years	TB rate per 100,000 person-years	95% CI
Total patients	446	3	1238.7	242.2	49.9, 708.8
Positive QFT	95	1	277.6	360.2	9.1, 2007.1
Negative or indeterminate QFT	351	2	961.1	208.1	25.2, 751.7
Living donor-recipient pairs	234	2	551.8	362.5	43.9, 1309.3
Recipient QFT (+)/Donor QFT (+)	16	0	51.6	0	0, 7149.9
Recipient QFT (+)/Donor QFT (−)	20	1	48.2	2074.7	52.5, 11559.4
Recipient QFT (−)/Donor QFT (+)	57	1	134.9	741.3	18.8, 4130.2
Recipient QFT (−)/Donor QFT (−)	141	0	317.1	0	0, 1163.3

*CI* confidence interval, *No* number, *QFT* QuantiFERON test, *TB* tuberculosis

When 234 KT recipients with donor QFT were evaluated, 2 active TB cases were noted, one from a recipient QFT (+)/donor QFT (−) pair and another from a recipient QFT (−)/donor QFT (+) pair (Fig. 1b). The incidences of TB in each situation are shown in Table 3.

The clinical characteristics of 3 patients with post-KT TB are summarized in Table 4. All cases were living-donor KT without desensitization, and post-KT times to TB diagnosis were 38, 10, and 7 months after KT. One patient had pulmonary TB, and 2 patients had extrapulmonary TB.

**Table 4:** Clinical characteristics of post-transplant tuberculosis cases

	Case 1	Case 2	Case 3
Age range	40 - 50	40 - 50	40 - 50
Previous treatment (duration)	Preemptive	HD (4 months)	HD (1 month)
LDKT or DDKT	LDKT	LDKT	LDKT
Recipient QFT	Negative	Positive	Negative
Donor QFT	Positive	Negative	N/A
Recipient TST	N/A	N/A	0 mm
Desensitization	No	No	No
Induction immunosuppression	Basiliximab	Basiliximab	Basiliximab
Maintenance immunosuppression	Steroid, tacrolimus, MMF	Steroid, tacrolimus, MMF	Steroid, tacrolimus, MMF
Time to TB diagnosis after KT	38 months	10 months	7 months
Type of TB	TB lymphadenopathy	TB colitis	Pulmonary TB

*F* female, *HD* hemodialysis, *KT* kidney transplantation, *LDKT* living-donor kidney transplantation, *M* male, *MMF* mycophenolate mofetil, *N/A*, not applicable, *TB* tuberculosis

### The predictive ability of QuantiFERON for post-transplant TB

We performed Poisson regression analysis to assess the predictive ability of the QFT for post-KT TB development (Table 5). The analysis of 446 recipients with QFT showed that QFT positivity did not predict post-KT TB development (RR, 1.847; 95% CI, 0.168–20.373; *P* = 0.616). When the 234 donor-recipient pairs were analyzed, neither recipient QFT positivity (RR, 5.012; 95% CI, 0.301–83.430; *P* = 0.261) nor donor QFT positivity (RR, 1.758; 95% CI, 0.106–29.274; *P* = 0.694) could predict post-KT TB development (Table 5).

**Table 5:** Poisson regression analysis for predictablity of QuantiFERON test for post-transplant tuberculosis

`	RR	95% CI	*P* value
Model 1			
Recipient QFT (+)	1.847	0.168, 20.373	0.616
Model 2			
Recipient QFT (+)	5.012	0.301, 83.430	0.261
Donor QFT (+)	1.758	0.106, 29.274	0.694

*CI* confidence interval, *QFT* QuantiFERON test, *RR* relative risk

A total of 446 recipients without clinical latent TB were included in model 1 and 234 donor-recipient pairs with QFT results were included in model 2

## Discussion

In this study, the QFT was positive in 22% of KT recipients, and recipient QFT positivity was associated with clinically latent TB before KT. We showed that that the incidence of post-KT TB was 0.242 cases/100 person-years among 446 KT recipients at a median follow-up of 30.2 months and that neither recipient QFT positivity nor donor QFT positivity could predict post-KT TB development.

Consistent with previous reports [12, 13], the QFT was significantly associated with latent TB, whereas the TST showed no such association in patients with ESRD. Moreover, agreement between the QFT and TST was poor. Overall, our study demonstrated that the QFT is a better diagnostic test for latent TB than TST in KT candidates. Treatment of latent TB is recommended for solid organ transplant recipients with a positive TST or IGRA result [2–4]. However, the actual benefit of this practice is still unknown [2, 14]. IGRA yields a high rate of false positives [15], and some TB breakthrough cases developed despite isoniazid prophylaxis [16, 17]. Moreover, concerns about drug toxicity and resistance may not justify routine isoniazid prophylaxis for recipients with a positive IGRA result in a country with high-to-intermediate TB burden, unless the predictive capacity of IGRA for post-TB development is confirmed.

Several studies have been performed to validate the diagnostic usefulness of the IGRA test for active TB. Pooled sensitivity for active TB was 80% for the QFT and 81% for the TB-specific ELISPOT assay (T-SPOT), and pooled specificity of the QFT for active TB was 79 and 59% for T-SPOT [18]. However, the diagnostic performance for extrapulmonary TB that is more common in KT patients [19, 20] was slightly lower; pooled sensitivity was 72% for the QFT and 90% for T-SPOT, and pooled specificity was 82% for the QFT and 68% for T-SPOT [21]. IGRA also showed a good negative predictive value (NPV) but a very poor positive predictive value (PPV) for progression to active TB [2]. Pre-transplant T-SPOT failed to show significant predictive potential for post-transplant TB development [9]. In parallel, the PPV and NPV of the QFT to predict TB in our results were 1.05 and 99.43%, respectively. Although the QFT seems to be slightly more specific than T-SPOT, our results showed that the QFT cannot sufficiently predict post-transplant TB development. Taken together, we propose using isoniazid prophylaxis for all patients with clinically latent TB. Regarding the cases of positive IGRA without evidence of clinically latent TB, routine isoniazid prophylaxis may not be needed for KT patients in intermediate to high TB-burden countries until we can obtain contradictory evidence from large-scale, long-term follow-up studies, due to a low PPV of IGRA for active TB development and concerns about drug toxicity and resistance.

Interestingly, two cases of TB developed in recipients with negative QFT results. This suggested that the QFT, just like T-SPOT, is not adequately sensitive in immunosuppressed hosts [9]. De novo post-KT TB infection can also occur in countries with a high-to-intermediate TB burden. Yet, latent TB in the donor may be transmitted to the recipient after KT [22]. Actually, one case of TB in a patient with a negative QFT result received kidneys from a QFT-positive donor. Organ donation from donors with active TB is generally contraindicated; however, there is some controversy concerning isoniazid treatment for donors or recipients when the donor has positive IGRA results. Some guidelines recommend chemoprophylaxis for donors with a positive TST/IGRA before donation [22]; however, it is challenging to treat all donors with positive IGRA results for the same reason as recipients. In our study, 31% of donors were QFT positive, and the QFT of the donor could not predict the development of TB. Although treatment of latent TB for KT recipients with QFT-positive donors is recommended in low TB-burden countries, routine treatment is still controversial in high-to-intermediate TB-burden countries, because drug toxicity and resistance may outweigh the benefits of treatment [22]. Further large-scale, long-term follow-up studies are needed to confirm the necessity of treatment based on donor QFT. Before confirmative evidence is available, a careful approach is needed according to a center-specific policy, and we recommend close monitoring of KT patients with a positive donor IGRA result without routine treatment in our situation.

There were some limitations to our study. First, our study was retrospective; therefore, the exact history of contact with active TB patients was difficult to ascertain. Second, the QFT or TST was not performed for all recipients. The QFT was preferentially performed in adult or living donor KT patients. However, when we analyzed the incidence rate of post-KT TB in patients at our center who did not undergo the QFT and in the study population of the KNOW-KT [23], there was no significant difference in the incidence rate between our study population and either group. Next, the follow-up duration was relatively short and the study population was small for the adequate assessment of the predictive usefulness of post-transplant TB development. Nevertheless, the present study is the first study to assess the predictive potential of pre-KT QFT in recipients and donors for post-KT TB development in an intermediate TB-burden country. Larger scale studies with a long-term follow-up are needed to verify our findings.

## Conclusions

The QFT is more useful than the TST for diagnosing a latent TB infection in KT candidates. However, the QFT in both recipients and paired living donors may not predict the short-term development of post-transplant TB in an intermediate-TB-burden country.

## Abbreviations

BCG: Bacillus Calmette-Guérin
CI: Confidence interval
ESRD: End-stage renal disease
IFN: Interferon
IGRA: Interferon-γ release assay
KT: Kidney transplantation
NPV: Negative predictive value
PPV: Positive predictive value
QFT: QuantiFERON TB Gold In-Tube test
RR: Relative risk
TB: Tuberculosis
T-SPOT: Tuberculosis-specific ELISPOT assay
TST: Tuberculin skin test
